# Type 2 Diabetes Reduces the Muscle Anabolic Effect of Resistance Exercise Training in Older Adults

**DOI:** 10.1093/geroni/igaa057.1706

**Published:** 2020-12-16

**Authors:** Amanda Randolph, Tatiana Moro, Adetutu Odejimi, Blake Rasmussen, Elena Volpi

**Affiliations:** The University of Texas Medical Branch at Galveston, Galveston, Texas, United States

## Abstract

Type 2 Diabetes Mellitus (T2DM) accelerates the incidence and increases the prevalence of sarcopenia in older adults. This suggests an urgent need for identifying effective sarcopenia treatments for older adults with T2DM. It is unknown whether traditional approaches, such as progressive resistance exercise training (PRET), can effectively counteract sarcopenia in older patients with T2DM. To test the efficacy of PRET for the treatment of sarcopenia in older adults with T2DM, 30 subjects (15 T2DM and 15 age- and sex- matched controls) underwent metabolic testing with muscle biopsies before and after a 13-week full-body PRET program. Primary outcome measures included changes in appendicular lean mass, muscle strength, and mixed muscle fractional synthesis rate (FSR). Before PRET, BMI-adjusted appendicular lean mass was significantly lower in the T2DM group (0.7095±0.0381 versus 0.8151±0.0439, p<0.0001). As a result of PRET, appendicular lean mass adjusted for BMI and muscle strength increased significantly in both groups, but to a lesser extent for the T2DM group (p=0.0009) . Preliminary results for FSR (n=25) indicate that subjects with T2DM had lower basal FSR prior to PRET (p=0.0197) . Basal FSR increased significantly in the control group after PRET (p=0.0196), while it did not change in the T2DM group (p=0.3537). These results suggest that in older adults the positive effect of PRET on muscle anabolism and strength is reduced by T2DM . Thus, older adults with T2DM may require more intensive, multimodal and targeted sarcopenia treatment. Funded by NIH R01AG049611 and P30AG024832.

